# Identifying metabolite markers for preterm birth in cervicovaginal fluid by magnetic resonance spectroscopy

**DOI:** 10.1007/s11306-016-0985-x

**Published:** 2016-03-08

**Authors:** Emmanuel Amabebe, Steven Reynolds, Victoria L. Stern, Jennifer L. Parker, Graham P. Stafford, Martyn N. Paley, Dilly O. C. Anumba

**Affiliations:** Academic Unit of Reproductive and Developmental Medicine, University of Sheffield, Sheffield, South Yorkshire UK; Integrated BioSciences, School of Clinical Dentistry, University of Sheffield, Sheffield, South Yorkshire UK; Academic Unit of Radiology, Department of Cardiovascular Science, University of Sheffield, Sheffield, South Yorkshire UK; Academic Unit of Reproductive and Developmental Medicine-Obstetrics and Gynecology, Department of Human Metabolism, University of Sheffield, 4th Floor, Jessop Wing, Tree Root Walk, Sheffield, S10 2SF UK

**Keywords:** Cervicovaginal fluid, Metabolites, Pregnancy, Preterm birth, Nuclear magnetic resonance (NMR)

## Abstract

**Introduction:**

Preterm birth (PTB) may be preceded by changes in the vaginal microflora and metabolite profiles.

**Objectives:**

We sought to characterise the metabolite profile of cervicovaginal fluid (CVF) of pregnant women by 1H NMR spectroscopy, and assess their predictive value for PTB.

**Methods:**

A pair of high-vaginal swabs was obtained from pregnant women with no evidence of clinical infection and grouped as follows: asymptomatic low risk (ALR) women with no previous history of PTB, assessed at 20–22 gestational weeks, g.w., n = 83; asymptomatic high risk (AHR) women with a previous history of PTB, assessed at both 20–22 g.w., n = 71, and 26–28 g.w., n = 58; and women presenting with symptoms of preterm labor (PTL) (SYM), assessed at 24–36 g.w., n = 65. Vaginal secretions were dissolved in phosphate buffered saline and scanned with a 9.4 T NMR spectrometer.

**Results:**

Six metabolites (lactate, alanine, acetate, glutamine/glutamate, succinate and glucose) were analysed. In all study cohorts vaginal pH correlated with lactate integral (r = −0.62, *p* < 0.0001). Lactate integrals were higher in the term ALR compared to the AHR (20–22 g.w.) women (*p* = 0.003). Acetate integrals were higher in the preterm versus term women for the AHR (20–22 g.w.) (*p* = 0.048) and SYM (*p* = 0.003) groups; and was predictive of PTB < 37 g.w. (AUC 0.78; 95 % CI 0.61–0.95), and delivery within 2 weeks of the index assessment (AUC 0.84; 95 % CI 0.64–1) in the SYM women, whilst other metabolites were not.

**Conclusion:**

High CVF acetate integral of women with symptoms of PTL appears predictive of preterm delivery, as well as delivery within 2 weeks of presentation.

**Electronic supplementary material:**

The online version of this article (doi:10.1007/s11306-016-0985-x) contains supplementary material, which is available to authorized users.

## Introduction

Preterm birth (PTB), defined as birth before 37 weeks of gestation, is the most notable cause of perinatal morbidity and mortality worldwide (Blencowe et al. 2013; Hamilton et al. 2013). Approximately 15 million PTBs (about 11.1 % of all births) occur annually (Blencowe et al. 2013), costing in excess of $26 billion to health care providers annually (MacDorman et al. 2007).

Approximately two-thirds of PTBs occur without an identifiable cause and follow spontaneous onset of preterm labor (PTL), whilst a third of cases are associated with preterm premature rupture of membranes (Goldenberg et al. 2008). About 25–40 % of PTBs are associated with intrauterine infection or inflammation (Goldenberg et al. 2008; Romero et al. 2001). Changes in the vaginal microflora have been implicated in the pathogenesis of ascending intrauterine infection (Racicot et al. 2013; Romero et al. 2006), and may be undetectable using conventional culture-dependent techniques (DiGiulio et al. 2008).

An approach to elucidating the host-microbial changes associated with PTB is to identify the metabolite changes associated with these interactions. Vaginal bacterial colonization and infection results in the release of lipopolysaccharides, peptidoglycans and short chain fatty acid, which evoke deciduitis and chorioamnionitis. Subsequently proinflammatory chemokines and cytokines such as TNF-α, IL1-α, IL-1β, IL-6, IL-8, and granulocyte colony stimulating factor, are synthesized and released (Goldenberg et al. 2000a; Kawai and Akira 2011; Mirmonsef et al. 2012; Schenten and Medzhitov 2011). These trigger a cascade of responses including the release of matrix metalloproteinases (e.g. MMP-8) (Rahkonen et al. 2009a; Witkin et al. 2013), and prostaglandins (e.g. PGE_2_ and PGF_2α_), (Goldenberg et al. 2000b; Romero et al. 2006), some of which degrade the extracellular matrix proteins of the cervix and chorioamniotic membrane causing cervical remodeling, rupture of the fetal membranes and myometrial contractions, leading to PTB (Goldenberg et al. 2000b; Romero et al. 2006; Witkin et al. 2013).

Measures to predict the risk of PTB are few, being confined to evaluation of a previous history of PTB, a short cervix (<25 mm) on second trimester ultrasonography (Crane and Hutchens 2008; Goldenberg et al. 2008; Mella and Berghella 2009), and the assessment of cervicovaginal fluid (CVF) for fetal fibronectin (Honest et al. 2009), and phosphorylated insulin-like growth factor binding protein-1 (phIGFBP-1) (Rahkonen et al. 2009b), amongst other analytes. Each of these tests has its limitations, the principal one being low positive predictive values (Di Renzo et al. 2011). The detection of clinical infection has not been shown to be predictive of PTB and data regarding the pathogenic role of bacterial vaginosis (BV) is unclear (Donders et al. 2010; Guaschino et al. 2006). A need subsists to identify new biomarkers that reliably predict PTB (Honest et al. 2006).

The assessment of metabolite markers of tissue and microbial anaerobic and aerobic glycolysis is finding increasing application in characterizing physiological and disease states (Auray-Blais et al. 2011; Ghartey et al. 2015; Horgan et al. 2009; Mamas et al. 2011). A recent study has identified differences in the metabolite profiles of women with BV compared to women without the infection (Srinivasan et al. 2015).

It is plausible that the cervicovaginal ecosystem of commensal and pathogenic organisms may generate metabolite markers that may predict the risk of PTL and PTB. A technique for assessing such metabolite changes is nuclear magnetic resonance (NMR) spectroscopy. Recently, NMR has been employed to show that vaginal secretions rich in species of *Lactobacillus* were associated with high amounts of lactate (Bai et al. 2012; Gajer et al. 2012). Additionally, acetate, butyrate, succinate and propionate levels were high in vaginal samples dominated by anaerobic bacteria such as *Atopobium*, *Megasphera*, *Parvimonas*, *Mobiluncus*, *Prevotella* etc. (Bai et al. 2012).

We therefore sought to characterize the metabolite profile of CVF obtained from cohorts of pregnant women who deliver prematurely compared to those who do not, categorizing the participants by whether they were at high-risk of PTB or not, and by presentation with symptoms of PTL. We hypothesized that the ^1^H NMR spectrum of CVF may reliably predict pregnant women who deliver prematurely, as well as women with symptoms of PTL who deliver shortly after assessment.

## Materials and methods

These studies were approved by the Yorkshire & Humber (Sheffield) Committee of the UK National Research Ethics Service (REC Number 13/YH/0167).

The study participants comprised of two clinical categories of pregnant women: those that had no symptoms of PTL (asymptomatic group) and those presenting to the delivery suite with symptoms of, but not established, PTL. The asymptomatic pregnant women were further classified into 2 gestationally-matched groups based on a previous history of PTB: a low-risk group (ALR), who had no previous history of PTB (assessed at 20–22 gestational weeks, g.w., n = 83), and a high-risk group (AHR), who had a previous history of PTB (n = 71, assessed at 20–22, and repeated at 26–28 g.w., n = 58). The third study group (SYM) comprised women presenting with clinical symptoms of PTL but who were not in established labor (infrequent uterine contractions less than thrice every 10 min, cervix <4 cm dilated, and intact fetal membranes, 24–36 g.w., n = 65). The SYM group was studied before any clinical therapy was initiated and women who were already on tocolytic or antibiotic drugs or any form of vaginal topical therapy were excluded from study. All participants were recruited via the antenatal clinics and Triage Delivery Suites of the Jessop Wing Hospital, Sheffield, UK.

At presentation, a pair of high vaginal samples was obtained with dry polystyrene Dacron swabs (Deltalab Eurotubo 300263, Fisher Scientific, UK) from each woman following written informed consent. The collected samples were immediately processed or stored in a refrigerator at −20 °C, for up to 3 days, pending analysis. The clinical course and delivery outcomes of participants were subsequently ascertained. Women with multiple gestation, bacteriologically proven infection, and history of abnormal cervical cytology within 3 years, ruptured fetal membranes, and prior vaginal examination at presentation were excluded from the study.

### ^1^H NMR sample preparation

The end of the swab soaked with the vaginal fluid sample was cut off and placed in a 1.5 ml microfuge tube. Six hundred microlitre of Phosphate Buffered Saline at pH 7.4 was added to the tube as an extraction solvent. The vaginal fluid was washed off the swab by vortexing for 5 min, after which the swab was removed and safely discarded. The solution was then centrifuged at 13,000 rpm for 3 min to separate swab particles from the vaginal fluid solution. The supernatant was carefully aspirated into a separate clean 1.5 ml microfuge tube and stored at −80 °C ready for analysis. Prior to ^1^H NMR analysis, a total of 400 µl of each sample comprising of 380 µl of vaginal fluid in solution and 20 µl of deuterium oxide (D_2_O) was transferred into a 5 mm NMR tube. An unused (sterile) polystyrene Dacron swab was also prepared as above and analyzed using the same protocol as a background signal control. This was repeated at regular intervals or change of swab batch to control for swab manufacturing variations.

### ^1^H NMR spectroscopy

Using a 9.4T Bruker Avance III NMR spectrometer (Bruker BioSpin GmbH, Karlsruhe, Germany), with 5 mm broadband observe probe, ^1^H NMR spectra were acquired using the Watergate water suppression pulse sequence (NS = 256, D1 = 5 s, AQ = 1 s, SW = 20.6 ppm, TD = 16,446), for each of the vaginal samples. All ^1^H NMR experiments were performed at approximately 21 °C. Data was acquired and processed using the Bruker Topspin 2.1.6 software to produce a phase and baseline corrected spectrum. All spectra were referenced to the ^1^H lactate signal at δ = 1.33 ppm. Identified metabolite signals in the ^1^H NMR spectra were integrated for peak area (which is proportional to metabolite concentration). To correct for differences in vaginal fluid concentration (variation in the swab sampling), each metabolite integral was divided by the total spectrum integral (δ = 0.0–10.0 ppm, excluding the residual water signal between δ = 4.7–5.0 ppm) to provide a normalized integral (N.I.) (Bai et al. 2012; Gajer et al. 2012).

### Assigning the NMR spectral peaks

In order to assign metabolites to the ^1^H NMR spectra, 2-D ^1^H-^1^H and ^1^H-^13^C NMR spectra were obtained. The following 2-D NMR spectra were acquired in order to assign metabolites to the ^1^H NMR spectral peaks: ^1^H-^13^C presat-HSQC (Heteronuclear Single Quantum Correlation spectroscopy)—NS = 1024, D1 = 1 s, AQ = 0.078 × 0.006 s, SW = 10.0 × 150 ppm, TD = 624 × 180; ^1^H-^13^C presat-HMBC (Heteronuclear Multiple Bond Correlation spectroscopy)—NS = 1024, D1 = 1 s, AQ = 0.128 × 0.005 s, SW = 10.0 × 200 ppm, TD = 1024 × 200; ^1^H-^1^H watergate-DQFCOSY (Double Quantum Filtered Correlation spectroscopy)—NS = 256, D1 = 0.5 s, AQ = 0.832 × 0.022 s, SW = 9.0 × 9.0 ppm, TD = 6000 × 160; and ^1^H-^1^H presat-cleanTOCSY (Total Correlation spectroscopy)—NS = 16, D1 = 1.5 s, AQ = 0.284 × 0.071 s, SW = 9.0 × 9.0 ppm, TD = 2048 × 512.

### Vaginal fluid pH and fetal fibronectin measurement

During vaginal fluid sample collection, the vaginal pH was also determined by obtaining a sample of vaginal discharge from the lateral vaginal wall with the aid of a dry swab and smeared on a narrow range pH paper (pH-Fix, Macherey–Nagel, DE, range 3.6–6.1).

Fetal fibronectin level was quantified using a bedside immunoassay—quantitative Rapid fFN 10Q analyzer (Hologic, Marlborough, MA).

### Polymerase chain reaction

Vaginal microbial DNA was extracted and amplified with QIAamp DNA mini kit (Qiagen, UK) and genus-specific primers targeted at the bacterial 16S rRNA gene (Supplementary Table S1), to examine for a range of commensal and potential pathogenic species (details in Supplementary File 1).

### Statistical analysis

All Statistical and ROC curve analysis were performed using MATLAB (Mathworks, Natick, MA), Both Wilcoxon rank-sum test and ANOVA, with Bonferroni PostHoc test were performed to compare differences in metabolite N.I. between and within the groups. Values are quoted as mean ± SE unless otherwise stated. The predictive potential of the vaginal fluid metabolites for PTB was determined by receiver operating characteristics (ROC) curves for the following comparisons:

- Preterm (<37 weeks) versus term births in all groups
- <32 versus >32 g.w. in symptomatic women
- <2 versus >2 weeks from presentation to delivery in symptomatic women

Predictive accuracy was also determined for metabolite N.I. as sensitivity, specificity, negative and positive predictive values, as well as likelihood ratios (Akobeng 2007; Altman and Bland 1994).

## Results

### Pregnancy outcomes

Table 1 summarizes the clinical details of the participant cohorts. Only 1.2 % of the ALR women delivered preterm (single birth). Prevalence of PTB in the AHR women was 36.6 and 29.3 % in the 20–22 and 26–28 g.w. groups respectively. Of women presenting with symptoms of PTL (SYM), 83.1 % went on to deliver at term, while 16.9 % delivered preterm. The mean time between presentation and delivery was 14.5 ± 4.4 and 61.6 ± 3.7 days for preterm and term-delivered SYM women respectively.

**Table 1:** Clinical characteristics of the study participants

Characteristics	Asymptomatic low risk women, 20–22 g.w.		Asymptomatic high risk women, 20–22 g.w.		Asymptomatic high risk women, 26–28 g.w.		Symptomatic women, 24–36 g.w.	
	Preterm^b^	Term	Preterm	Term	Preterm	Term	Preterm	Term
Age (years)	29	29.6 ± 0.5 (19–39) 82	32.4 ± 1.1 (19–45) 25	30.9 ± 0.8 (19–39) 44	31.4 ± 1.1 (24–39) 17	30.1 ± 0.9 (19–40) 39	*29.4* *±* 2.4* *(22–48)* *11*	*26.4* *±* *0.8* *(16–44)* *54*
BMI (kg m^−2^)	26.3	25.9 ± 0.6 (14.7–42.2) 82	28.5 ± 1.3 (18.2–46.5) 25	27.0 ± 0.8 (18.2–40.4) 44	27.5 ± 1.3 (18.2–34.1) 17	28.5 ± 0.8 (18.2–40.4) 39	26.6 + 0.9^a^ (23.1–30.2) 7	25.4 ± 0.7^a^ (17.4–41.6) 48
Previous history of PTB (n)	NA	NA	18	35	12	30	NA	NA
Cervical length (mm)	37	*41.0* *±* 0.8** *(29–66)* *82*	*28.4* *±* *2.4* *(13–53)* *21*	*36.4* *±* 1.2* *(18–49)* *39*	*24.2* *±* *2.6* *(8–40)* *17*	*34.6* *±* 1.4* *(15–50)* *37*	26.8 ± 7.5^a^ (9–45) 4	32.4 ± 1.7^a^ (14–54) 25
Fetal fibronectin conc. (ng/ml)	4	*16.3* *±* 3.2** *(1–187)* *81*	*100.9* *±* *29.5* *(2–453)* *21*	29.4 ± 6.2 (2–157) 37	*131.1* *±* 35.4* *(3–442)* *16*	*18.6* *±* *5.7* *(1–159)* *35*	190.5 ± 185^a^ (6–375) 2	15.7 ± 3.9^a^ (2–74) 26
Gestational age at study (days)	142	141 ± 0.6 (132–159) 82	146 ± 2.0 (115–165) 26	147 ± 1.7 (103–175) 45	189 ± 1.5 (182–205) 17	188 ± 1.0 (173–203) 41	209 ± 6.4 (187–257) 11	213 ± 3.5 (139–254) 54
Gestational age at birth (days)	238	281 ± 1.0 (258–299) 82	223 ± 6.6 (146–253) 25	274 ± 1.3 (260–290) 45	235 ± 5.1 (187–253) 16	275 ± 1.4 (260–295) 41	224 ± 7.5 (187–257) 11	276 ± 1.2 (260–295) 50
Vagina pH	4.1	*3.9* *±* 0.04** *(3.6–5)* *82*	*4.3* *±* *0.1* *(3.6–5.3)* *21*	*4.2* *±* *0.1* *(3.6–5.6)* *38*	*4.2* *±* *0.2* *(3.6–6.1)* *17*	4.1 ± 0.1 (3.6–4.7) 37	4.0 ± 0.2^a^ (3.6–4.4) 3	4.3 ± 0.1^a^ (3.6–6.1) 25
Prevalence of PTB, %	1.2		36.6		29.3		16.9	

Data are presented as mean ± SE (range) n

Italics indicate groups with significant differences

*g.w*. gestational weeks, *BMI* body mass index, *PTB* preterm birth, *NA* not applicable

* Differences between term and preterm-delivered women within the group, *p* < 0.05

** Differences between term-delivered ALR women and the AHR (20–22 g.w.) women, *p* ≤ 0.01

^a^Low study participants population (N) due to absence of consent

^b^Single member of cohort

### Treatment interventions

A subset of the AHR group had undergone clinical therapy by the time of their assessment at 20–22 g.w. (cervical cerclage 12 %; progesterone 8 %; betamethasone 2 % and antibiotics, 40 %), and at 26–28 g.w. (cervical cerclage 13 %; progesterone 23 %; betamethasone 11 %; and antibiotics 70 %).

### Cervical length and fetal fibronectin concentration

The term-delivered ALR had significantly longer cervical lengths (Table 1), compared to the term (*p* = 0.01) and preterm-delivered (*p* < 0.00001) women in the AHR (20-22 g.w.) group. Similar term vs preterm differences were observed within the AHR (20-22 g.w.) (*p* = 0.002), and AHR (26–28 g.w.) groups (*p* = 0.004).

The ALR women that delivered at term had significantly lower fetal fibronectin levels (Table 1), compared to the preterm AHR (20–22 g.w.) women (*p* < 0.001). A similar result in relation to delivery outcome was observed within the AHR (26–28 g.w.) group (*p* < 0.001).

No differences in cervical length and fetal fibronectin level were observed in the SYM group.

### NMR integration

**Fig. 1:**
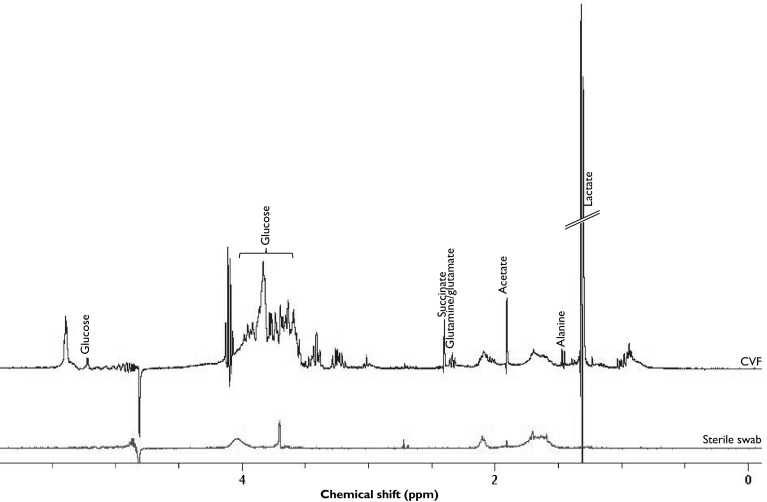
One dimensional ^1^H NMR spectrum for metabolites observed in cervicovaginal fluid (CVF) samples and sterile swab (*red*). *ppm* parts per million (Color figure online)

**Fig. 2:**
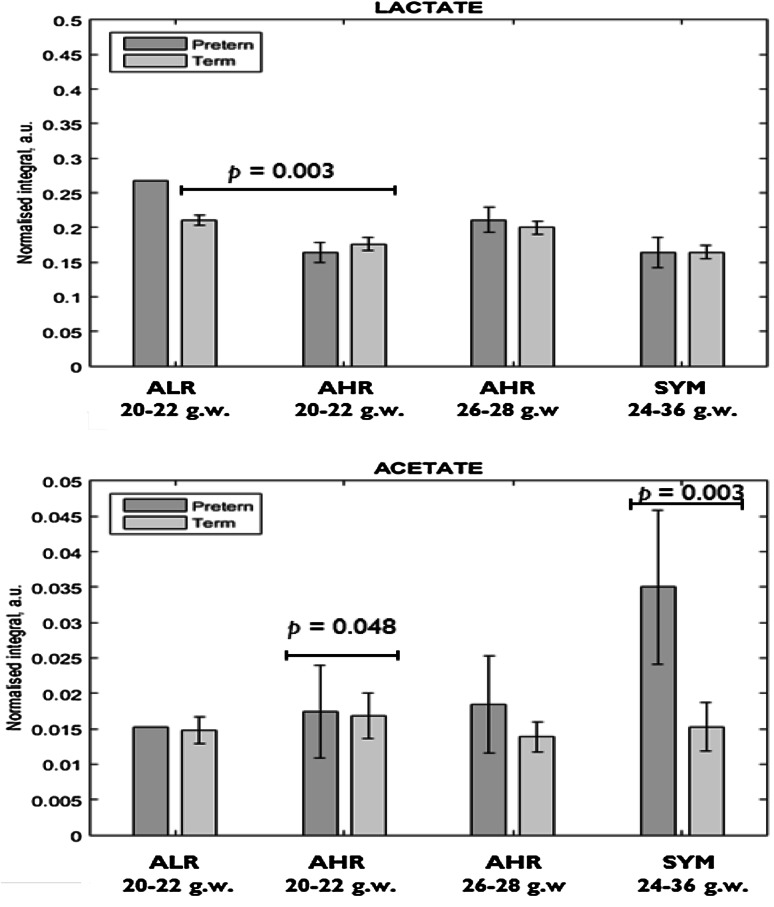
Data are presented as mean ± SE. Only one preterm delivery was recorded in the asymptomatic low risk group. *ALR* asymptomatic low risk women, *AHR* asymptomatic high risk women, *SYM* symptomatic women, *g.w*. gestation weeks. *p value <0.05 considered statistically significant (Color figure online)

Six metabolite signals of interest were identified in the ^1^H NMR spectra (Fig. 1). These were lactate, alanine, acetate, glutamine/glutamate, succinate, and glucose. These metabolites were analysed due to their association with the vaginal microbiota and impact on vaginal pH (Srinivasan et al. 2015, Aldunate et al. 2015). The glucose signal at δ = 5.2 ppm was integrated and used in subsequent analysis to avoid influence from other peaks in the glucose signal region of 3.2–3.9 ppm. Comparison of mean ^1^H metabolite N.I. from the different cohorts in relation to delivery outcomes is shown in Fig. 2 and Supplementary Fig. S1. There were no differences in the ^1^H NMR total spectrum absolute integrals, indicating that vaginal fluid concentration did not vary significantly between the groups (Supplementary Fig. S2).

### Lactate

The lactate N.I. of the term ALR women was significantly higher than that of the term and preterm AHR (20–22 g.w.) women (*p* = 0.003, ANOVA). The lactate N.I. did not differ between AHR and SYM women who delivered preterm and their respective counterparts who delivered at term.

### Acetate

The acetate N.I. was higher in the preterm than the term group for the SYM (*p* = 0.003) (about twofold) and AHR (20–22 g.w.) women (*p* = 0.048). Acetate N.I. was not significantly different in other study cohorts in relation to delivery gestation.

### Alanine

There was a non-significant trend to higher alanine N.I. in the preterm-delivered SYM women in relation to their term counterparts (*p* = 0.056). The alanine N.I. did not differ significantly between preterm and term-delivered women in the AHR groups.

### Succinate, glucose and glutamate/glutamine

The SYM women who delivered preterm showed a non-significant trend to higher succinate N.I. compared to their term counterparts (*p* = 0.08). The AHR (20–22 g.w.) women who delivered at term had a non-significant higher glutamine/glutamate N.I. compared to their preterm-delivered counterparts (*p* = 0.09).

No differences in glucose N.I. were observed in any cohorts in relation to delivery outcomes.

No significant differences in the metabolite N.I. were observed between the AHR (26–28 g.w.) who delivered at term versus preterm.

### Vaginal pH

**Fig. 3:**
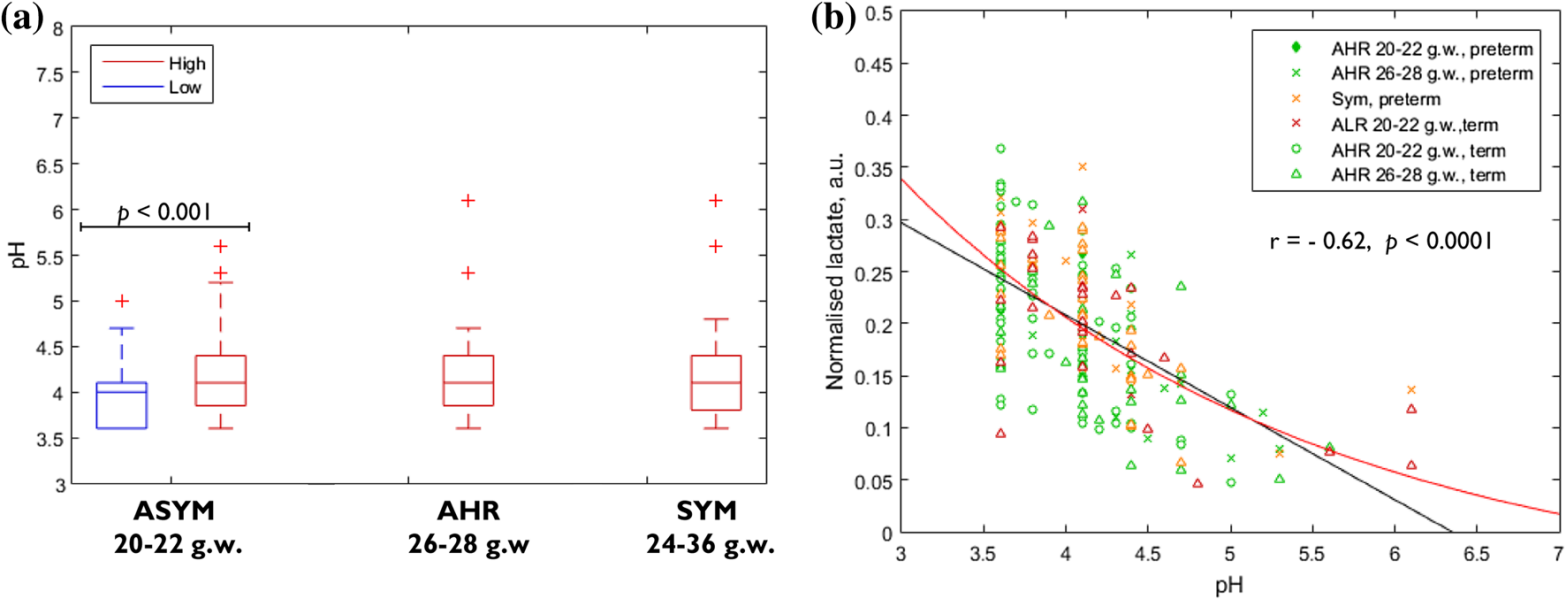
**a** Cervicovaginal fluid pH between the different cohorts. *Box plots* show the median line, with the box edges representing the 25 and 75 % quartiles. *Whiskers* extend to the furthermost value within 1.5 times the interquartile range from 25 to 75 % quartiles. **b** Vaginal fluid pH values versus normalized lactate integral, preterm and term groups. The correlation was virtually identical for all groups irrespective of delivery outcome, demonstrating that lactate contribute the most to pH. Black line represents a linear fit, r = −0.62, *p* < 0.0001. *Red line* represents a log_10_ fit. pH range 3.6–6.1. *ASYM* asymptomatic women (*ALR* asymptomatic low risk and AHR 20–22 g.w. women) *AHR* asymptomatic high risk women, *SYM* symptomatic women, *g.w*. gestational weeks (Color figure online)

Significantly lower vaginal pH was observed in ALR women compared to the AHR (20–22 g.w.) women (*p* < 0.001). There was a significant negative correlation between vaginal fluid lactate N.I. and pH (r = −0.62, *p* < 0.0001) (Fig. 3a, b).

### Predictive performance of metabolites for preterm birth

**Fig. 4:**
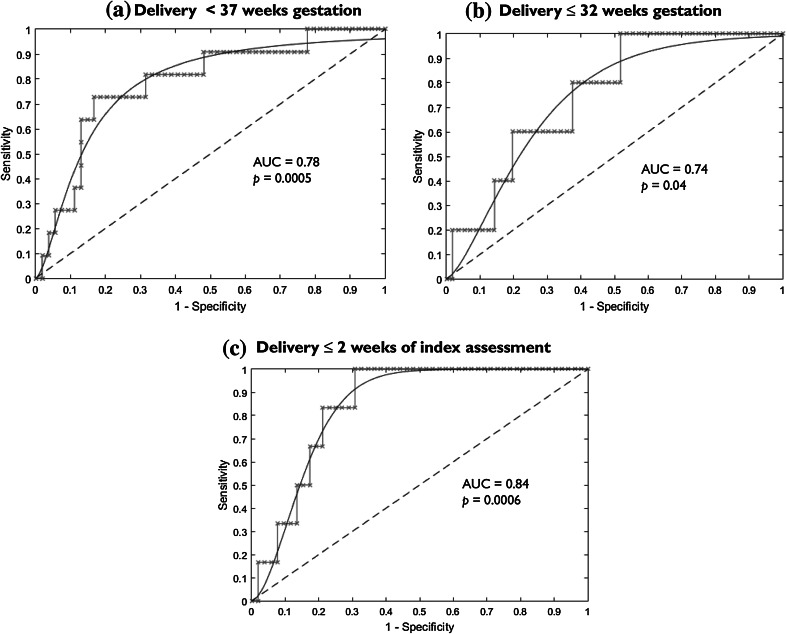
Performance of acetate integrals in predicting preterm delivery in symptomatic pregnant women. **a** <37 g.w. **b** ≤32 g.w. **c** Within 2 weeks of index assessment. *AUC* area under the ROC curve

Analysis of the area under the ROC curves for the study metabolites showed that the acetate N.I. was highly predictive of women presenting with symptoms of PTL who delivered prematurely (<37 and <32 g.w.), and within 2 weeks of the index assessment (Table 2; Fig. 4). The other metabolites did not show predictive potential for PTB for any study cohorts.

**Table 2:** Predictive performance of acetate normalised integrals for preterm birth in symptomatic pregnant women

Variables	<37 weeks gestation	≤32 weeks gestation	≤2 weeks of index assessment
Area under the ROC curve (AUC)	0.78	0.74	0.84
Standard error, SE	0.09	0.13	0.1
95 % confidence interval	0.61–0.95	0.48–0.99	0.64–1.00
Significance level, *p*	0.0005	0.04	0.0006
Youden index, J	0.83	0.48	0.69
Sensitivity, %	73	100	100
Specificity, %	83	48	69
Positive predictive value (PPV), %	47	15	27
Negative predictive value (NPV), %	94	100	100
Positive likelihood ratio, LR+	4.4	1.9	3.3
Negative likelihood ratio, LR−	0.2	0.5	0.3

### PCR detection of important vaginal organisms

Qualitative PCR assessment of microbial composition of the vaginal microbome revealed the presence of higher prevalence of potentially pathogenic anaerobes (i.e. *Gardnerella*, *Fusobacterium*, *Bacteroides*, *Mobiluncus*, and *Mycoplasma* species) in the preterm-delivered women compared to term counterparts especially in the SYM women (Supplementary Table S2*).*

## Discussion

We have studied six potential metabolite markers of PTB using ^1^H NMR (Fig. 1), in the two clinical cohorts that pose prediction challenges in clinical practice: asymptomatic high-risk pregnant women (who are at risk of recurrence) and women presenting with symptoms of PTL which are often spurious and may not ultimately lead to preterm delivery. We describe the variation in normalized integrals in a subset of CVF metabolites of pregnant women judged to be at low or high risk of PTB on the basis of past history across mid/late gestations, as well as those women who present with symptoms of PTL.

Our pilot observations showed significant predictive ability for CVF acetate in SYM women who delivered prematurely before 37, as well as before 32 weeks, and within 14 days of presentation. Restricting the criteria for prematurity to birth before 32 weeks or within 14 days of presentation reduced the PPV and NPV from the ROC analysis due to the smaller size of the cohorts. Nonetheless, the lower PPVs and NPVs were still comparable to those of the current predictive markers for PTB used in clinical practice i.e. fetal fibronectin, phIGFBP-1 and cervical length (Heng et al. 2015). The limited number of women in these smaller cohorts who delivered preterm highlights the need for larger studies to confirm or refute these findings. Confirmation of our observations could enable better stratification of pregnant women and optimization of their clinical care. The high negative predictive values attained in our series also suggest that low acetate N.I. may prove a good “rule-out” test for imminent premature birth, enabling women to be discharged to outpatient care and thus providing maternal reassurance. Whether CVF acetate will improve the prognostic performance of currently-employed assessment modalities such as ultrasound cervical length (Crane and Hutchens 2008; Mella and Berghella 2009), and fetal fibronectin (Honest et al. 2009), remains to be determined in adequately-powered prospective studies.

The nature and origin of the CVF metabolites is unclear. Their profile is thought to result from host mucosal-microbial metabolism of sugars and proteins within that milieu. Indeed changes in vaginal bacterial community composition have been linked to changes in the metabolite signature (Gajer et al. 2012), and have been implicated in ascending intrauterine infection (Romero et al. 2001, 2006), leading to spontaneous PTL (Andrew et al. 2000; Romero et al. 2001). This is a plausible explanation of our finding of a link between acetate, higher prevalence proportions of mixed anaerobes (i.e. *Gardnerella*, *Fusobacterium*, *Bacteroides*, *Mobiluncus*, *Mycoplasma*) and PTB. However, data on vaginal microbial communities of pregnant women is conflicting, with some studies failing to demonstrate any difference between women who deliver preterm and term (Romero et al. 2014).

We have observed elevated CVF lactate N.I. associated with lower pH in the low-risk asymptomatic cohort consisting of pregnant women with no history of PTB. Interestingly, only one woman in this group delivered preterm in our series. In contrast, the vaginal fluid lactate N.I. of the higher risk AHR and SYM cohorts were lower, with correspondingly higher vaginal fluid alkalinity (Table 1; Fig. 3a), consistent with the long-established observation that these changes are characteristic of BV, a risk factor for PTB (Beghini et al. 2014; Donders et al. 2009; Klebanoff et al. 2005; Lamont and Taylor-Robinson 2010). Supporting this view we observed a higher prevalence of anaerobic organisms in the preterm-delivered symptomatic women compared to their term counterparts (Table S1). It has been postulated that large amounts of acetate produced by vaginal anaerobes may contribute to the pro-inflammatory environment associated with preterm labour and birth (Chaudry et al. 2004; Mirmonsef et al. 2012; Romero et al. 2004). Furthermore, increased vaginal acetate is a feature of infection by the anaerobic pathogenic species seen in BV (Al-Mushrif et al. 2000; Chaudry et al. 2004; Mirmonsef et al. 2012), consistent with our findings that SYM women who delivered preterm and/or within 2 weeks of presentation had greater than twofold higher acetate, lower lactate N.I.s as well as higher prevalence of plausibly subclinical colonization with BV-associated species. This is also supportive of the unproven thesis that reduced acidity of CVF, resulting from a change in the microflora from the normal commensal pattern predominated by *Lactobacillus* species to a more diverse community with increased anaerobic species, may play a role in inflammation-induced PTB. It is plausible that when the bacterial flora responsible for the much higher acetate N.I. observed in the preterm SYM attain a functional metabolic threshold they may well provide a ‘trigger’ for preterm delivery. However larger prospective studies, as well as studies with a functional mechanistic design, will be required to explore this thesis.

A recent study employed liquid and gas chromatography mass spectrometry (GCMS) techniques to highlight that the CVF metabolite fingerprint is altered several weeks before preterm compared to term delivery (Ghartey et al. 2015). Our findings employing NMR at similar gestational time points support this finding. Additionally, we have pinpointed elevated CVF acetate N.I., not accurately demonstrable using GCMS techniques owing to the relative volatility of acetate (Srinivasan et al. 2015), in women presenting at later gestations with symptoms of PTL who deliver preterm. Also, we observed a non-significant trend to higher glutamine/glutamate N.I. in the AHR 20–22 g.w. women who delivered at term compared to their preterm-delivered counterparts. Glutamate levels identified by GCMS is negatively correlated with elevated vaginal pH and *L. iners* dominated microflora (Srinivasan et al. 2015), which has been associated with intermediate vaginal flora, BV and PTB (Elovit et al. 2014; MacKlaim et al. 2013; Petricevic et al. 2014).

It is of note that several vaginal anaerobes produce succinate from glycogen. However, although there was a trend towards increased succinate N.I. in the CVF of women presenting with symptoms of PTL who delivered prematurely, this was not statistically significant, indicating that this mechanism may not be highly active in the bacterial communities of our cohorts. Succinate is produced by anaerobic fermentation of glycogen during infection (Al-Mushrif et al. 2000; Chaudry et al. 2004; Gajer et al. 2012;), by *Prevotella* and *Mobiluncus* (Al-Mushrif et al. 2000; Donders et al. 2002; Graham et al. 2013), species that we detected in more of the preterm-delivered SYM women (Supplementary Table S2), contributing to the non-significant trend observed.

Our data in relation to alanine N.I. is somewhat variable and unclear. Vaginal microflora produce alanine through transamination of pyruvate (Chen et al. 1982), and increased alanine concentrations has been reported in CVF in non-specific vaginitis and BV states (Chen et al. 1982), in which the fluid is rich in amino acids and peptides due to the ascendency of proteolytic organisms such as *P. bivia*, *G. vaginalis*, *Mobiluncus* (Eschenbach 1989; McGregor et al. 1986; Paavonen 1983; Thomason et al. 1988; Schoonmaker et al. 1991). The latter organisms produce succinate and acetate (Pybus and Onderdonk 1997, Al-Mushrif et al. 2000), and elevate vaginal pH (Aldunate et al. 2015).

We did not observe any significant differences in the metabolite N.I. between the AHR (26–28 g.w.) who delivered at term versus preterm, and in comparison to other cohorts. Although the explanation for this observation is unclear, a proportion of women in this cohort received clinical interventions (such as cervical cerclage, and treatment with progesterone or antibiotics) and it may be speculated that these could have altered metabolite expression levels and underlines the problems inherent in clinical studies of PTB. All women studied in all cohorts had conventional care and ethical constraints precluded any studies that withheld routine clinical care from any study participants. To avoid clinical interventions confounding our results, and in order to bypass this problem, we studied the SYM group before any clinical intervention had been commenced. The preterm-delivered women in this group had altered metabolite profiles compared to their term-delivered counterparts and were studied at a mean later gestation than the AHR and ALR cohorts. The AHR group were studied at two earlier gestational time points as part of their routine clinical follow-up protocol at our hospital. It is plausible that had they been studied again at later gestations comparable to the SYM group significant differences in metabolite profiles may have been noted. Future studies will be required involving larger cohort sizes to elucidate the influence of such interventions as tocolytic, antibiotic, progesterone, and steroid therapy on the vaginal metabolome. To bypass ethical limitations such studies will need to employ a logistic regression prediction model to control for confounding variables.

Our study identifies promising applications of cervicovaginal organic acid assays (particularly acetate) for prognosticating PTB. Our findings also suggest that understanding the metabolite signature of pregnant women may clarify the pathogenesis of inflammation-induced PTB. However the study has several limitations. We have so far identified only a limited number of metabolites and further studies on larger numbers of parturients and metabolites (e.g. d/l-lactate ratios), under controlled physiological conditions, will be required to elucidate a metabolite signature of PTB. The dynamic nature of the vaginal ecosystem (Gajer et al. 2012; Ravel et al. 2011, 2013; Srinivasan et al. 2010), and individual variations in CVF volume, may influence study observations. However, our standardization of sample preparation, inclusion of ‘mock’ sterile swabs and the normalization of the metabolite integrals to the total spectrum integral have largely negated these potential variations.

The causes of PTB are multi-factorial and the correlation of the metabolite profile with new or existing prognostic assessments may enhance predictive ability. Going forward, absolute quantification of metabolites would facilitate potential clinical translation of metabolite assays but would present several methodological challenges. Furthermore, we have studied participants at specific gestational time points and it is plausible that the expression of metabolites change across the full gestational time spectrum in a way that was not observed in this study. A larger and more diverse cohort with stricter inclusion/exclusion criteria could confirm and strengthen our findings and allow stratification of the cohorts in relation to the risk of PTB.

## Concluding remarks

We have reported high acetate N.I. and increased detection of potentially pathogenic anaerobes, in the CVF of pregnant women presenting with symptoms of PTL who ultimately deliver before 37 weeks and within 2 weeks of assessment. Understanding the mechanisms and functional implications of these observations, as well as determining whether quantitative CVF acetate has clinically useful predictive potential for PTB, requires further investigation in larger studies.

## Electronic supplementary material

Heatmap of relative abundance of vaginal fluid metabolites of pregnant women by 1H NMR. Lactate normalized integral is divided by 10. ALR, asymptomatic low risk women; AHR, asymptomatic high risk women; SYM, symptomatic women; g.w., gestation weeks.Supplementary material 1 (PPTX 1809 kb)

Comparison of 1H NMR total spectrum absolute integrals of vaginal fluid in the various cohorts in relation to pregnancy outcomes indicated no significant difference. Box plots show the median line, with the box edges representing the 25% and 75% quartiles. Whiskers extend to the furthermost value within 1.5 times the interquartile range from the 25% and 75% quartiles.ALR, asymptomatic low risk women; AHR, asymptomatic high risk women; SYM, symptomatic women; g.w., gestational weeks.Supplementary material 2 (PPTX 750 kb)

Supplementary material 3 (DOCX 21 kb)
