# Supplementary material for: Identifying metabolite markers for preterm birth in cervicovaginal fluid by magnetic resonance spectroscopy
Source: Metabolomics. 2016 Mar 8;12:67. doi: 10.1007/s11306-016-0985-x (PMC4783437; doi:10.1007/s11306-016-0985-x)
Supplement: Supplementary file 1 — Heatmap of relative abundance of vaginal fluid metabolites of pregnant women by 1H NMR. Lactate normalized integral is divided by 10. ALR, asymptomatic low risk women; AHR, asymptomatic high risk women; SYM, symptomatic women; g.w., gestation weeks.Supplementary material 1 (PPTX 1809 kb) [file 11306_2016_985_MOESM1_ESM.pptx]

## Slide 1
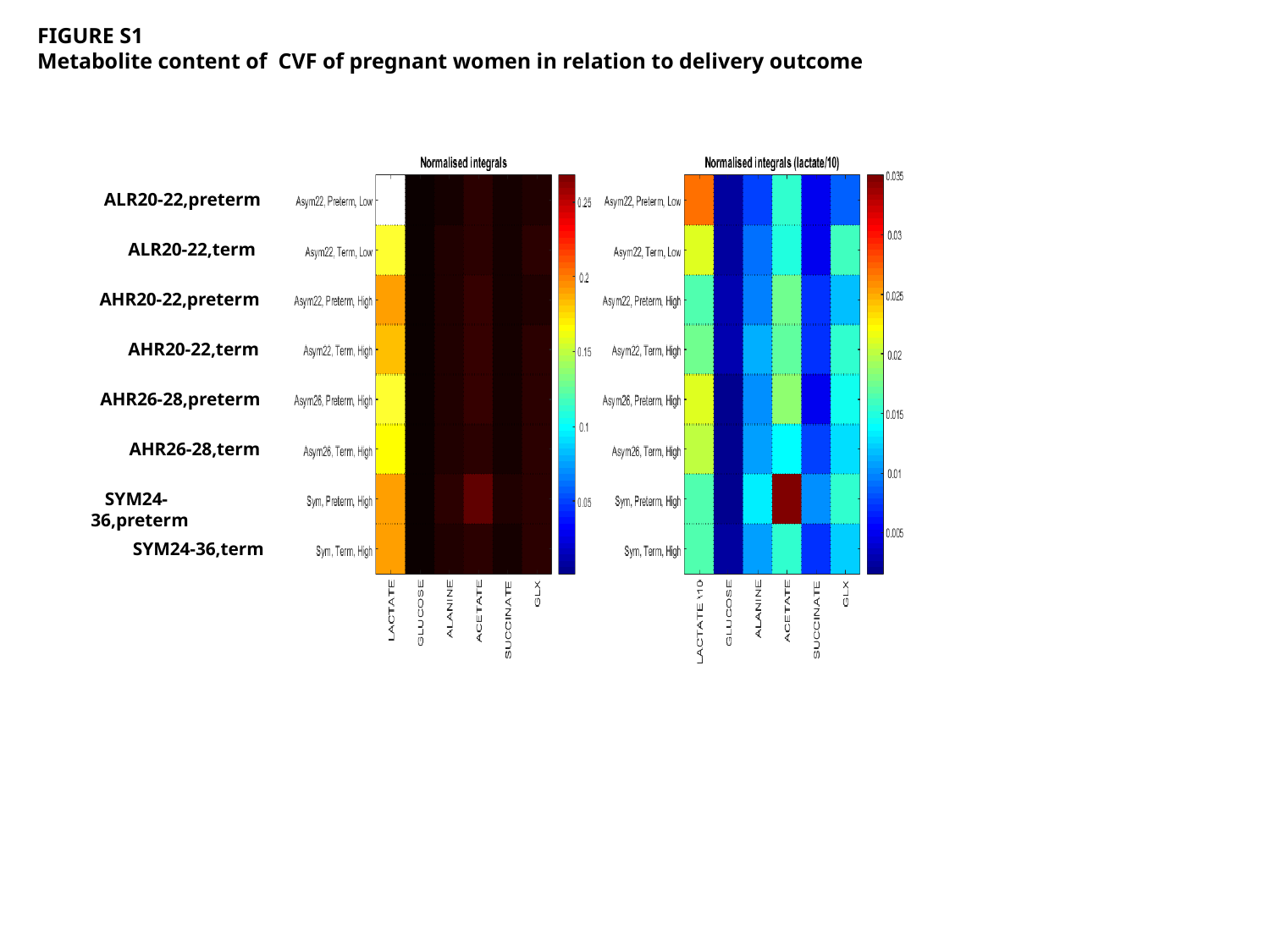

FIGURE S1
Metabolite content of CVF of pregnant women in relation to delivery outcome
 ALR20-22,preterm
 ALR20-22,term
 AHR20-22,preterm
 AHR20-22,term
 AHR26-28,preterm
 AHR26-28,term
 SYM24-36,preterm
 SYM24-36,term
