# Supplementary material for: Identifying metabolite markers for preterm birth in cervicovaginal fluid by magnetic resonance spectroscopy
Source: Metabolomics. 2016 Mar 8;12:67. doi: 10.1007/s11306-016-0985-x (PMC4783437; doi:10.1007/s11306-016-0985-x)
Supplement: Supplementary file 2 — Comparison of 1H NMR total spectrum absolute integrals of vaginal fluid in the various cohorts in relation to pregnancy outcomes indicated no significant difference. Box plots show the median line, with the box edges representing the 25% and 75% quartiles. Whiskers extend to the furthermost value within 1.5 times the interquartile range from the 25% and 75% quartiles.ALR, asymptomatic low risk women; AHR, asymptomatic high risk women; SYM, symptomatic women; g.w., gestational weeks.Supplementary material 2 (PPTX 750 kb) [file 11306_2016_985_MOESM2_ESM.pptx]

## Slide 1
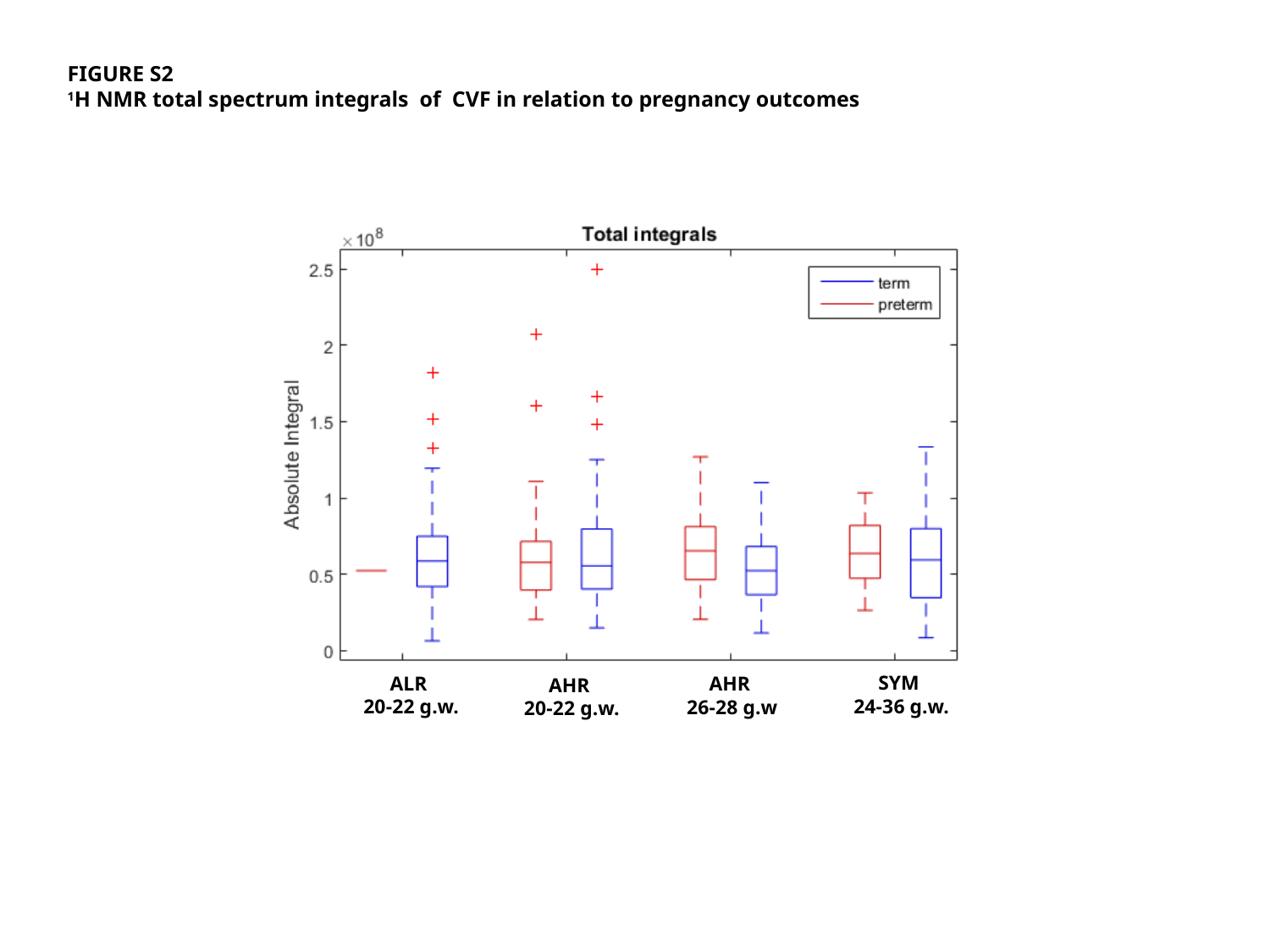

FIGURE S2
1H NMR total spectrum integrals of CVF in relation to pregnancy outcomes
SYM
 24-36 g.w.
ALR
 20-22 g.w.
AHR
26-28 g.w
AHR
 20-22 g.w.
