# Supplementary material for: Identifying metabolite markers for preterm birth in cervicovaginal fluid by magnetic resonance spectroscopy
Source: Metabolomics. 2016 Mar 8;12:67. doi: 10.1007/s11306-016-0985-x (PMC4783437; doi:10.1007/s11306-016-0985-x)
Supplement: Supplementary file 3 — Supplementary material 3 (DOCX 21 kb) [file 11306_2016_985_MOESM3_ESM.docx]

PCR amplification

Bacterial genus-specific primers (Sigma-Aldrich, UK), targeted at the bacterial 16S rRNA gene were used to examine for a range of commensal and potentially pathogenic species which included *Lactobacillus*, *Bacteroides* (*Prevotella*), *Gardnerella*, Group B *Streptococcus*, *Fusobacterium*, *Mycoplasma* and *Mobiluncus*. The names of the primers, their sequences, targets, annealing temperatures, amplicon product size and sources are represented in *Supplementary* Table S1. PCR amplification experiments were performed using 12.5 µl AmpliTaq Gold DNA polymerase (Applied Biosystems, Thermo Fisher Scientific, UK ), 5 ng genomic DNA template, 1 µl each of forward and reverse primers in a total reaction volume of 25 µl. Reactions were run in an Applied Biosystems 2720 Thermal cycler (Life Technologies, UK) with the following cycling criteria: 95°C (5 mins) – denaturation, followed by 35 cycles of 95°C (1 min) - denaturing, 50 - 62°C depending on the primer sets (1 min) – annealing, 72°C (1 min) – elongation, with a final extension at 72°C (7 mins). The results were visualized on a UV-transilluminator by agarose gel electrophoresis and ethidium bromide staining. Positive results were assigned according to the presence or absence of bands of the appropriate size.

**Table S1**

**Bacterial genus-specific primers, targets, and annealing temperatures**

| Primer^a^ | Sequence (5’-3’) | Target | Annealing temp (ºC) | Amplicon  Size  (bp) | Reference |
| --- | --- | --- | --- | --- | --- |
| LABF  LABR | AGAGTTTGATYMTGGCTCAG  CACCGCTACACATGGAG | *Lactobacillus* | 62 | 667 | Ling et al. 2010 |
| FBF  FBR | ACTCCTACGGGAGGCAGCAGT  CGAATTTCACCTCTACACTTGT | *Fusobacterium* | 60 | 341 | Walter et al. 2002 |
| GV1F  GV3R | GGAAACGGGTGGTAATGCTGG  CGAAGCCTAGGTGGGCCATT | *G. vaginalis* | 55 | 125 | Zozaya-Hinchliffe et al. 2010 |
| BAC32F  BAC708R | AACGCTAGCTACAGGCTT  CAATCGGAGTTCTTCGTG | *Bacteroides-Prevotella* | 53 | 676 | Bernhard et al. 2000 |
| M. curt-440F  M. curt-1026R | TTCTCGCGAAAAAGGCACAG  CTGGCCCATCTCTGGAACCA | *M. curtisii* | 57 | 586 | Fredricks et al. 2007 |
| Mobil-577F  M.mulie-1026R | GCTCGTAGGTGGTTCGTCGC  CCACACCATCTCTGGCATG | *M. mulieris* | 62 | 449 | Fredricks et al. 2007 |
| Mh1-F  Mh2-R | CAATGGCTAATGCCGGATACGC  GGTACCGTCAGTCTGCAAT | *M. hominis* | 62 | 334 | Zariffard et al. 2002 |

^a^ Primers designed to detect the species-specific regions of the 16S rRNA gene.

**Table S2**

**Percentage of women in experimental cohorts expressing common vaginal bacterial species identified by PCR**

| Bacterial sp., % | Asymptomatic  Low risk women,  20-22 g.w. | | Asymptomatic  High risk women,  20-22 g.w. | | Asymptomatic  High risk women,  26-28 g.w. | | Symptomatic  women  24-36 g.w. | |
| --- | --- | --- | --- | --- | --- | --- | --- | --- |
|  | Preterm | Term  N=44 | Preterm  N=19 | Term  N=29 | Preterm  N=6 | Term  N=18 | Preterm  N=10 | Term  N=50 |
| *Lactobacillus*^a^ | NA | 100 | 100 | 100 | 100 | 100 | 100 | 100 |
| *Fusobacterium*^b^ | NA | 13 | 16 | 16 | 14 | 10 | 27^*^ | 9 |
| *Gardnerella vaginalis*^b^ | NA | 96 | 100 | 94 | 100^*^ | 80 | 100^*^ | 87 |
| *Bacteroides-Prevotella*^b^ | NA | 26 | 16 | 23 | 43^*^ | 25 | 64^*^ | 49 |
| *Mobiluncus curtisii*^b^ | NA | 30 | 26 | 29 | 29 | 20 | 27 | 36 |
| *Mobiluncus mulieris*^b^ | NA | 9 | 0 | 3 | 14 | 10 | 18^*^ | 2 |
| *Mycoplasma hominis*^b^ | NA | 2 | 11 | 3 | 0 | 0 | 36^*^ | 11 |

*N*, number of vaginal fluid samples; %, percentage of bacterial sp. in samples.

^a^ Commensal bacterial sp. (major lactate producer).

^b^ Potentially pathogenic bacterial sp. associated with abnormal vaginal microflora, infection and PTB (produce high amounts of acetate and succinate).

^*^ Differences in the prevalence of bacterial sp. between term and preterm-delivered women ≥ 10%.
